# p75^NTR^, but Not proNGF, Is Upregulated Following Status Epilepticus in Mice

**DOI:** 10.1177/1759091414552185

**Published:** 2014-09-24

**Authors:** Melissa W. VonDran, John LaFrancois, Victoria A. Padow, Wilma J. Friedman, Helen E. Scharfman, Teresa A. Milner, Barbara L. Hempstead

**Affiliations:** 1 Department of Medicine, Weill Cornell Medical College, New York, NY, USA; 2 Center of Dementia Research, The Nathan Kline Institute for Psychiatric Research, Orangeburg, NY, USA; 3 Department of Biological Sciences, Rutgers Life Sciences Center, Rutgers University, Newark, NJ, USA; 4 Brain and Mind Research Institute, Weill Cornell Medical College, New York, NY, USA; 5 Laboratory of Neuroendocrinology, The Rockefeller University, New York, NY, USA

**Keywords:** growth factors, proneurotrophins, BDNF, NGF, p75 neurotrophin receptor, sorCS2

## Abstract

ProNGF and p75^NTR^ are upregulated and induce cell death following status epilepticus (SE) in rats. However, less is known about the proneurotrophin response to SE in mice, a more genetically tractable species where mechanisms can be more readily dissected. We evaluated the temporal- and cell-specific induction of the proneurotrophins and their receptors, including p75^NTR^, sortilin, and sorCS2, following mild SE induced with kainic acid (KA) or severe SE induced by pilocarpine. We found that mature NGF, p75^NTR^, and proBDNF were upregulated following SE, while proNGF was not altered, indicating potential mechanistic differences between rats and mice. ProBDNF was localized to mossy fibers and microglia following SE. p75^NTR^ was transiently induced primarily in axons and axon terminals following SE, as well as in neuron and astrocyte cell bodies. ProBDNF and p75^NTR^ increased independently of cell death and their localization was different depending on the severity of SE. We also examined the expression of proneurotrophin co-receptors, sortilin and sorCS2. Following severe SE, sorCS2, but not sortilin, was elevated in neurons and astrocytes. These data indicate that important differences exist between rat and mouse in the proneurotrophin response following SE. Moreover, the proBDNF and p75^NTR^ increase after seizures in the absence of significant cell death suggests that proneurotrophin signaling may play other roles following SE.

## Introduction

Neurotrophins are a family of growth factors that include nerve growth factor (NGF), brain-derived neurotrophic factor (BDNF), neurotrophin-3 (NT-3), and neurotrophin-4 (NT-4). Neurotrophins have well-defined roles in the peripheral nervous system and central nervous system (CNS) with effects on cell survival, differentiation, and synaptic plasticity (Barde, 1994; Bibel and Barde, 2000; Huang and Reichardt, 2001; Chao, 2003). The actions of neurotrophins are mediated by two distinct receptor classes: the Trk family of tyrosine kinase receptors that selectively bind to the individual neurotrophins and the p75 neurotrophin receptor (p75^NTR^) that binds to all of the neurotrophins with low affinity. The well-described effects of neurotrophins on survival, differentiation, and synaptic plasticity are mediated by the Trk receptors (Huang and Reichardt, 2003), whereas the biological effects mediated by p75^NTR^ are more pleiotropic (Barker, 2004; Teng and Hempstead, 2004).

Neurotrophins are initially synthesized as precursors that can be secreted as proneurotrophins, which have distinct functions from their cleaved products (Lee et al., 2001; Mowla et al., 2001). Proneurotrophins bind with high affinity to p75^NTR^ and can act in complex with sortilin or sorCS2, members of the Vps10p family of receptors, to induce apoptosis or influence synaptic plasticity both in culture and *in vivo* (Teng et al., 2010; Yang et al., 2014; Koshimizu et al., 2009). The roles for neurotrophins in the brain are thus dependent on the form of neurotrophin that is expressed, as well as the receptors that are present, which may change during development or following neural injury.

Neurotrophins and their receptors have been implicated in the development of epilepsy (epileptogenesis) using multiple animal models (Binder et al., 2001; Friedman, 2010; McNamara and Scharfman, 2012). Thus, BDNF and NGF mRNA and protein are elevated following experimentally induced seizures, while NT-3 mRNA is unchanged or reduced (Ernfors et al., 1991; Isackson et al., 1991; Bengzon et al., 1992; Rudge et al., 1998). There is strong evidence from rodent models that the mature neurotrophins, NGF and BDNF, can promote epileptogenesis (Adams et al., 1997; McNamara and Scharfman, 2012). Recent studies also suggest a role for proneurotrophins in hippocampal cell death following status epilepticus (SE), when neuronal cell death typically occurs. One reason for the cell death after SE is likely to be the effects of proneurotrophins acting through p75^NTR^, as proBDNF and proNGF are upregulated in the rat hippocampus following SE induced by pilocarpine (Volosin et al., 2008). In addition, delivery of proNGF antibodies reduces the cell death present in the hilar region of the hippocampus following pilocarpine-induced SE (Volosin et al., 2008), suggesting that proNGF induction may be a mechanism contributing to the cell death following SE in the rat. P75^NTR^ also is upregulated in the hippocampus following SE and is associated with dying neurons (Roux et al., 1999; Volosin et al., 2008).

Most studies evaluating the effects of proneurotrophins on cell death after SE have been done in rats. The advantage of evaluating mice is the ease with which they can be genetically manipulated, which makes them invaluable tools. We were interested in determining whether the roles for proNGF observed in rats following SE could be extended to mice. Therefore, we investigated the temporal- and cell-specific induction of the proneurotrophins and their receptors to kainic acid (KA)- and pilocarpine-induced SE in mice. KA and pilocarpine are widely used chemoconvulsants that can induce SE and subsequently epilepsy in rodents (Ben-Ari, 1985; Turski et al., 1989; Leite et al., 2002; Pitkanen et al., 2006), although their mechanisms of action are distinct. KA acts as a glutamate receptor agonist while pilocarpine acts as a muscarinic receptor agonist, both of which result in limbic seizures. In rats, these models provide a consistent model for temporal lobe epilepsy (TLE), with hippocampal cell death resembling the pattern of cell loss in TLE, other hallmarks of TLE such as mossy fiber sprouting, and the development of spontaneous recurrent seizures. However, several strains of mice are resistant to epilepsy following chemoconvulsant injection (Schauwecker, 2011), making the study of epilepsy more difficult in this species. In our study, KA and pilocarpine resulted in varying degrees of seizure severity in mice. The degree of cell death in mice was correlated with the severity of SE, with KA eliciting mild, brief SE and limited cell death and pilocarpine eliciting severe, longer SE and widespread cell death in the hippocampus. These differences allowed us to investigate the proneurotrophin response following seizures that elicited cell death or not.

In contrast to rats, proBDNF, but not proNGF, was elevated in mice following both chemoconvulsants. We found proBDNF and p75^NTR^ were elevated following SE independent of cell death. Also, we report differences in p75^NTR^ localization following KA or pilocarpine. P75^NTR^-immunoreactivity (ir) was elevated in fibers in the hippocampus following both KA and pilocarpine but was associated with neurons and astrocytes only following pilocarpine. Sortilin did not change following SE, but sorCS2, a related sortilin family member and p75^NTR^ co-receptor, was elevated in neurons and astrocytes following pilocarpine-induced SE. These data indicate that some neurotrophin responses to seizures occur independent of cell death. In addition, these data suggest that there may be potential mechanistic differences in the neurotrophin response to SE between mice and rats that may be useful in developing therapies to target proneurotrophins and p75^NTR^.

## Materials and Methods

### Animals

Wild-type (+/+) and knock-in male or female mice expressing a hemagglutinin (HA)-epitope tagged NGF (NGF-HA) on a 129SvJ background were bred and maintained in our animal facility. NGF-HA knock-in mice were generated by substituting one allele of the murine coding exon of the NGF gene with the murine coding exon containing an HA-epitope tag added to the C-terminus as described previously (Siao et al., 2012). These mice have no mutation in NGF; they simply have an HA tag. Experimental protocols involving animals were reviewed and approved by the Weill Cornell Medical College Institutional Animal Care and Use Committee.

### Seizure Induction

For KA-induced seizures, 8- to 10-week-old wild-type (+/+) or NGF-HA mice were injected intraperitoneally (ip) with KA (25 mg/kg; Milestone Pharmtech, New Brunswick, NJ). For pilocarpine-induced seizures, mice were pretreated with 5 mg/kg methylscopolamine (subcutaneously, sc), a muscarinic receptor antagonist, to reduce the peripheral effects of pilocarpine. They were also treated with 50 mg/kg phenytoin (ip), an antiseizure drug that reduces mortality, 30 min prior to pilocarpine injection (320 mg/kg; ip). This protocol has been used by other investigators (Volosin et al., 2008). Both KA and pilocarpine result in continuous, repetitive limbic seizures (SE) if administered at a sufficient dose. The doses of KA and pilocarpine were chosen because they elicited the most severe seizures without significant mortality. Behavioral rating of seizures used the Racine scale (Racine, 1972). Stage 1 was characterized by behavioral arrest; Stage 2 by head nodding, gnawing, and mild tremors; Stage 3 by unilateral forelimb clonus; Stage 4 by bilateral forelimb clonus; and Stage 5 by severe seizures with prolonged loss of postural control or prolonged tonus. Animal behavior was closely observed following injection and for the duration of SE. The onset of SE was defined as the time when animals experienced continuous Stage 5 seizures. Mice that did not fulfill these criteria for SE were not included in analyses. Control animals received the same treatments, but saline was substituted for KA or pilocarpine. After 2 hr of SE, mice were treated with a dose of diazepam to reduce seizure severity (5–10 mg/kg). Control animals received the same dose of diazepam as experimental animals so that effects we analyzed were independent of effects of diazepam. During recovery, mice were injected with 0.5 ml Hartman’s solution (130 mM NaCl, 4 mM KCl, 3 mM CaCl, 28 mM lactate; 0.5 ml) subcutaneously twice daily until they were able to eat and drink ad libitum.

### Electroencephalographic (EEG) Recordings

Mice (6- to 8-week-old) were anesthetized with isoflurane and placed in a stereotaxic apparatus (David Kopf). After a midline incision, a bipolar 75-µm-diameter twisted stainless steel wire (A&M Systems) was positioned in each dorsal hippocampus (2 mm posterior to Bregma, 2 mm lateral to the midline, 1.5 mm deep). Two jeweler’s screws (Braintree Scientific) were positioned in the skull, one over left frontal cortex (1 mm posterior to Bregma and 2 mm lateral to the midline) and one over right occipital cortex (1 mm anterior to Lambda and 2 mm lateral to the midline). A 6-pin holder (Braintree Scientific) was centered over the skull with dental cement (Grip cement). The skin around the cement was swabbed with Betadine. The animal was placed under a heat lamp until fully ambulatory. Buprenorphine (Henry Schein) was administered sc (0.05 mg/kg) once, and once again after 12 hr.

After 2 weeks, animals were attached to a commutator in a standard mouse cage with overhead video camera (Axis Systems) and recorded at 2 kHz (Sirenia Software; Pinnacle Systems). After 20 min, they were injected with KA (25 mg/kg) or pilocarpine (250 mg/kg) as described earlier and recorded for 24 hr. The dosage of pilocarpine was lower than the dose used for animals without EEG electrodes because we found that higher doses led to mortality. The sample size for each condition was seven mice.

### Antibodies

The goat polyclonal antibody to p75^NTR^ and sheep polyclonal antibody to sorCS2 used for immunohistochemistry were purchased from R&D Systems (Minneapolis, MN), and their specificity was validated using p75^NTR^ or sorCS2 knockout tissue (data not shown). The mouse monoclonal antibody to the prodomain of BDNF was purchased from GeneCopoeia (Rockville, MD). The rabbit polyclonal Iba1 antibody was purchased from Wako Chemicals (Richmond, VA). The rabbit polyclonal glial fibrillary acidic protein (GFAP) antibody and the mouse monoclonal HA.11 antibody were purchased from Covance (Princeton, NJ). The rabbit polyclonal BDNF, NGF, and NeuN antibodies were purchased from Santa Cruz (Dallas, TX). The rabbit polyclonal p75^NTR^ antibody (9992) was generated in our laboratory (Esposito et al., 2001). The mouse monoclonal sortilin antibody was purchased from BD Biosciences (San Jose, CA). The mouse monoclonal tubulin antibody and the rabbit polyclonal HA antibody were purchased from Sigma-Aldrich (St. Louis, MO).

### Quantitative Light Microscopic Immunohistochemistry

Mice were anesthetized with tribromoethanol (240 mg/kg; Avertin, Sigma Aldrich) and perfused transcardially with saline followed by 4% paraformaldehyde in phosphate-buffered saline (PBS). The brains were removed and postfixed in 4% paraformaldehyde/PBS overnight and cryoprotected in 30% sucrose/PBS overnight. Horizontal sections (30 µm thick) were cut on a cryostat (Leica) and stored free floating in cryoprotectant solution (30% sucrose and 30% ethylene glycol in PB) at −20°C.

Sections were processed for immunoperoxidase using previously described methods (Hsu et al., 1981). Briefly, sections were washed in 0.1 M Tris buffer (TB), treated with 1% H_2_O_2_ diluted in TB for 30 min, and then placed in TB. Sections were processed through a series of incubations: (a) 0.1% Triton in TB, 10 min; (b) 0.005% bovine serum albumin (BSA) and 0.1% Triton in TB, 10 min; (c) 10% donkey serum, 0.005% BSA, and 0.1% Triton in TB, 1 hr; (d) incubation in anti-p75^NTR^ (1:800) or anti-sorCS2 (1:500) diluted in 0.005% BSA and 0.1% Triton in TB overnight at 4°C; (e) incubation with biotinylated anti-goat (for p75^NTR^) or anti-sheep (for sorCS2) secondary immunoglobulin (IgG; 1:400; Jackson ImmunoResearch) diluted in 0.005% BSA and 0.1% Triton in TB; and (f) incubation with avidin-biotin peroxidase complex (ABC kit; Vector) diluted in 0.005% BSA and 0.1% Triton in TB, 1 hr. All incubations were separated by 0.1% Triton/TB washes. After washing in PBS, control and experimental sections were developed identically at the same time using a VIP substrate kit for peroxidase (Vector).

Images of immunostained sections of the hippocampus were captured and analyzed at 100 or 400 × using a Zeiss microscope and camera (Obzerver.Z1 microscope, AxioCamMrm camera, Zeiss). For p75^NTR^ immunostaining, three mice were used per condition at 24 hr, 3 days, and 7 days post SE. For sorCS2 immunostaining, three mice were used per condition at 3 days post SE. For quantitative densitometry, regions of interest (ROI) were outlined, and the mean gray value was measured using Image J. To control for background staining in each section, the mean gray value of an area of the same size as the ROI with no labeling was subtracted. For each animal, six sections 120-µm apart through the mid-hippocampus were analyzed, and a total of three animals per condition were included in the analysis.

### Immunofluorescence Dual Labeling

Free-floating sections (30 µm thick) were washed in PBS and then treated with 1% NaBH_4_ diluted in PBS for 30 min. Next, sections were blocked with 3% donkey serum, 3% BSA, and 0.1% Triton in PBS for 1 hr followed by primary antibodies, proBDNF (1:200) + NeuN (1:500), proBDNF (1:200) + Iba1 (1:200), p75^NTR^ (1:750) + NeuN (1:500), sorCS2 (1:500) + NeuN (1:500), sorCS2 (1:500) + GFAP (1:1,000), 24 to 48 hr at 4°C. Sections were washed with PBS followed by incubation with Cy3 or Alexa Fluor-488-conjugated secondary IgGs (1:1,000; Invitrogen) for 1 hr. Images were captured and analyzed at 100 or 400 × (Obzerver.Z1 microscope, AxioCamMrm camera, Zeiss). The sample size was three mice per condition at 3 days post KA or pilocarpine.

### TUNEL and p75^NTR^ Dual Labeling

TUNEL (terminal deoxynucleotidyl transferase-mediated UTP nick end labeling) was performed using the DeadEnd Fluorometric TUNEL System (Promega) following the manufacturer’s protocol. Sections were then blocked with 3% donkey serum, 3% BSA, and 0.1% Triton in PBS for 1 hr followed by anti-p75^NTR^ (1:800) for 24 hr at 4°C. Sections were washed with PBS followed by incubation with Cy3-conjugated secondary IgG (1:1,000; Invitrogen) for 1 hr. Images were captured and analyzed at 100 or 400 × (Obzerver.Z1 microscope, AxioCamMrm camera, Zeiss). The sample size was three mice per condition at 3 days post SE.

### FluoroJade-B Staining

Degenerating neurons following seizures were evaluated in sections of the hippocampus by labeling with FluoroJade-B (Histo-Chem, Jefferson, AK), a polyanionic fluorescein derivative, following the manufacturer’s instructions. Images were captured and analyzed at 100 × (Obzerver.Z1 microscope, AxioCamMrm camera, Zeiss). All FluoroJade-B-positive cells in the hippocampus were manually counted using the Image J cell counter. The average number of cells/section was calculated from 12 serial sections separated by 120 µm from each animal. The sample size was three mice per condition at 24 hr, 3 days, and 7 days post SE.

### Immunoelectron Microscopy

Mice were processed for electron microscopic immunoperoxidase localization of p75^NTR^, as previously described (Milner et al., 2011). Briefly, mice were anesthetized with sodium pentobarbital (150 mg/kg, ip) and perfused transcardially with 3.75% acrolein and 2% paraformaldehyde in PB. The brains were removed and postfixed in 1.87% acrolein/2% paraformaldehyde for 30 min. Coronal sections (40 µm thick) were cut on a Leica vibrating microtome and stored in cryoprotectant solution (30% sucrose and 30% ethylene glycol in PB) at −20°C.

Prior to immunohistochemistry, sections were rinsed in PB, coded with hole punches, and pooled in containers to ensure identical processing (Pierce et al., 1999). Sections were incubated in 1% NaBH_4_ in PB for 30 min and rinsed in PB. Next, sections were transferred to TB and blocked with 0.5% BSA in TB for 30 min. Sections were incubated in anti-p75^NTR^ (1:800) in 0.1% BSA and 0.025% Triton in Tris saline (TS) for 4 days at 4°C. Sections then were incubated with biotinylated anti-goat IgG (1:400; Jackson ImmunoResearch) diluted in 0.1% BSA in TS followed by ABC at half the recommended dilution (Vector) for 30 min. Sections were reacted in 3,3′-diaminobenzidine (DAB) and 3% H_2_O_2_ in TS for 6 min and rinsed in TS followed by PB.

Sections were fixed in 2% osmium tetroxide in PB for 1 hr, dehydrated in increasing concentrations of ethanol and propylene oxide before being embedded in EmBed 812 (Electron Microscopy Sciences (EMS)) between two sheets of Aclar plastic (Milner et al., 2011). Ultra-thin sections (70–72 nm thick) were cut from area CA3 on a Leica UCT ultratome and collected on 400 mesh thin-bar grids (EMS). The grids were counterstained with uranyl acetate and Reynold’s lead citrate.

Sections from CA3 were examined at 11,500 × on a Tecnai transmission electron microscope (FEI) equipped with an Advanced Microscopy Techniques digital camera. p75^NTR^-ir profiles were identified and categorized using standard morphological criteria (Peters et al., 1991). Dendritic profiles contained regular microtubular arrays and were usually postsynaptic to axon terminal profiles. Unmyelinated axon profiles were smaller than 0.2 µm in diameter, contained a few synaptic vesicles, and lacked a synaptic junction in the plane of section. Axon terminal profiles had numerous small synaptic vesicles and had a cross-sectional diameter greater than 0.2 µm. Mossy fiber terminals could be distinguished by their large size (>1.0 µm) and their complex form including the envelopment of spinous profiles (Blackstad and Kjaerheim, 1961). When the identity of the p75^NTR^-ir profile could not be ascertained, it was categorized as *unknown*.

Single ultrathin sections taken from the surface of CA3 were examined from three mice from each experimental condition at 3 days. Micrographs containing p75^NTR^ labeled profiles were collected in random fields of stratum lucidum and stratum radiatum by a person blind to experimental condition. Criteria for field selection included good morphological preservation, the presence of immunolabeling in the field, and proximity to the plastic–tissue interface. In stratum lucidum 950 µm^2^ of neuropil and in stratum radiatum 500 µm^2^ of neuropil were sampled per animal.

### Western Blot and Immunoprecipitation

Dissected hippocampi were lysed in lysis buffer containing 1 × TB, 1% NP-40, 1% Triton-100, 10% glycerol, 1 mM phenylmethylsulfonyl fluoride (PMSF), and protease inhibitor cocktail (Sigma). Supernatants were collected following centrifugation at 14,000 × g. For immunoblot, 80 µg of total protein were run on polyacrylamide gels and transferred overnight. Following transfer, Western blots were fixed with 2.5% glutaraldehyde (for BDNF and NGF); blocked with 5% milk/Tris-buffered saline + Tween-20 (TBS-T); and incubated with anti-BDNF (1:1,000), anti-NGF (1:1,000), anti-p75^NTR^ (9992, 1:1,000), anti-sortilin (1:1,000), or anti-sorCS2 (1:1,000) for 2 hr. Blots were developed using HRP-conjugated secondary antibodies and an ECL kit (Amersham). To evaluate total protein levels, blots were stripped and reprobed with anti-tubulin (1:10,000; Sigma). Western blots were analyzed using Image J. For Western blots, sample sizes of 3 to 10 mice per condition were used at 24 hr, 3 days, and 7 days.

For HA immunoprecipitation, 1 mg of total protein from hippocampal lysates was incubated with a polyclonal HA antibody overnight at 4°C. Protein A-Sepharose beads (Sigma) were then added to the lysates for 1.5 hr at 4°C, and immunoprecipitates were collected by centrifugation for 5 min and 5,000 × g. Immunoprecipitates were washed 4 times with lysis buffer and run on a polyacrylamide gel. Following overnight transfer, Western blots were fixed with 2.5% glutaraldehyde, blocked with 5% milk/TBS-T, and incubated with anti-HA.11 (1:1,500) for 2 hr. Blots were developed using HRP-conjugated secondary antibodies and an ECL kit (Amersham). The sample size was three mice at 3 days KA and pilocarpine.

### Data Analysis

For each experiment, mice injected with KA or pilocarpine were compared with control injected with saline for the same timepoints. Statistical analysis was performed with StatView software, and the data are presented as mean ± SEM. Statistical differences were determined using Student’s *t* test or one-way analysis of variance (ANOVA) followed by Bonferroni, as appropriate. Conditions were considered significant at *p* < .05.

## Results

### KA Results in Limited Neuronal Death in the Hippocampus, Whereas Pilocarpine Results in Widespread Neuronal Death

**Figure 1.**
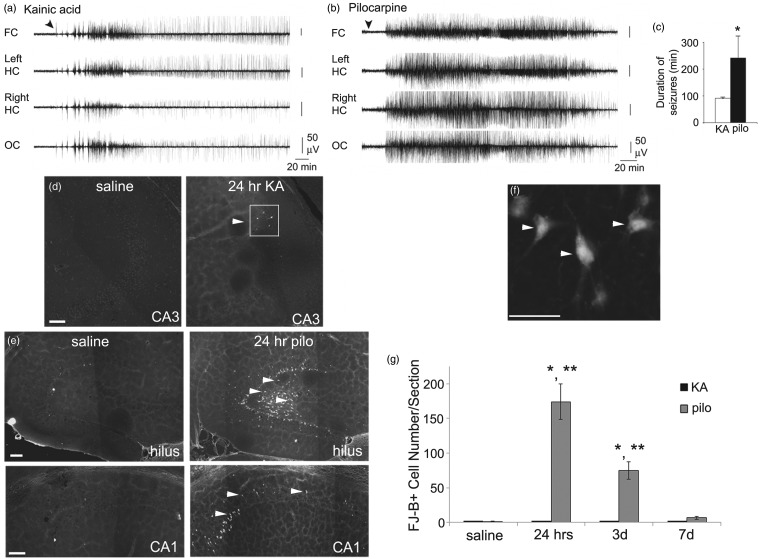
KA-injected mice exhibit mild SE without substantial hippocampal cell death, whereas pilocarpine-injected animals exhibit severe SE and widespread cell death. A cohort of mice were implanted with electrodes prior to injection of KA or pilocarpine and recorded for 24 hr after injection. (a–b) Representative EEG recordings from mice injected with KA (25 mg/kg) (a) or pilocarpine (250 mg/kg) (b). KA-injected mice showed a shorter period of intense EEG activity with prolonged EEG spiking afterwards. Pilocarpine-injected mice showed a prolonged period of intense EEG activity without later EEG spiking. FC = frontal cortex; HC = hippocampus; OC = occipital cortex. Calibrations in (a) are also for (b). (c) The duration of the acute period of EEG seizure activity was longer for pilocarpine-treated mice compared with KA-treated mice (*N* = 7/group, Student’s *t* test, *p* < .05). Duration was measured from the time of injection to the end of the last EEG seizure activity. Thus, pilocarpine-induced seizures were more severe than KA-evoked seizures. (d–e) Horizontal sections of hippocampus from mice injected with saline, KA (25 mg/kg), or pilocarpine (320 mg/kg) were processed for FJ-B staining. Representative images 24 hr following injection with saline, KA (d), or pilocarpine (e) are shown. White arrowheads indicate FJ-B-positive cells. The box in (d) outlines all of the FJ-B-positive cells in that section. Scale bar represents 100 µm. (f) A higher magnification image of FJ-B-positive cells from the hilus 24 hr following pilocarpine injection. White arrowheads indicate cells that are FJ-B-positive. Scale bar represents 50 µm. (g) All FJ-B-positive cells in the hippocampus were counted, and the average numbers of cells/section are shown for 24 hr, 3 days, and 7 days following injection of KA or pilocarpine. Data were analyzed by one-way ANOVA followed by Bonferroni. *Significantly different from saline controls (*p* < .05). ** Significantly different from KA (*p* < .05). *N* = 3 at each time point.

ProNGF as well as proBDNF are upregulated in the hilus following pilocarpine-induced SE in rats, and proNGF has been shown to play a role in the cell death following SE (Volosin et al., 2008). We were interested in determining if proNGF may also play a role in SE-induced cell death in mice. It is known that different inbred strains of mice have varying degrees of susceptibility to cell death following seizures (Schauwecker, 2012). Therefore, we first evaluated seizure activity and the induction of hippocampal cell death in the 129SvJ mouse strain following KA or pilocarpine. A cohort of animals was implanted with electrodes to measure neural activity by electroencephalography. KA (25 mg/kg) or pilocarpine (250 mg/kg) was injected, and mice were observed for behavioral signs of seizures following the Racine scale as well as recorded by EEG. All KA- and pilocarpine-injected mice had behavioral signs of SE, but the severity differed between the two chemoconvulsants. Mice injected with KA had robust electrographic seizures for only a brief period of time compared with pilocarpine-injected mice (Figure 1(a) to (c)). Therefore, seizures were less severe in response to KA compared with pilocarpine.

To evaluate cell death in the hippocampus, mice were sacrificed 24 hr, 3 days or 7 days after KA (25 mg/kg) or pilocarpine (320 mg/kg) injection. These animals did not have implanted electrodes, and the chemoconvulsant doses were adjusted so that the most severe SE was elicited without mortality. Tissue was processed for FluoroJade-B (FJ-B) to detect primarily degenerating neurons (Schmued and Hopkins, 2000). KA resulted in only a small number of FJ-B-positive cells in area CA3 of the hippocampus (Figure 1(d)), in only a subset of KA-injected mice. In contrast, pilocarpine resulted in a large number of FJ-B-positive cells in the dentate gyrus hilus and area CA1 in the hippocampus (Figure 1(e)), consistent with the typical pattern of cell loss after pilocarpine-induced SE in the rat (Volosin et al., 2008). As seen at higher power, the FJ-B-positive cells appeared to be neurons by morphology (Figure 1(f)). When total numbers of FJ-B-positive cells were quantitated, we found that KA resulted in no significant change in FJ-B cell number following 24 hr, 3 days, or 7 days, whereas pilocarpine resulted in a significant increase in FJ-B-positive cells at 24 hr and 3 days (Figure 1(g)). The limited cell death could be due to relatively mild SE after KA compared with pilocarpine. The observed differences between KA and pilocarpine injection in the 129SvJ mouse strain gave us an opportunity to evaluate the neurotrophin response following seizures with varying severity of SE and hippocampal damage.

### BDNF and Mature NGF Levels Are Elevated in the Hippocampus Following Both KA and Pilocarpine

We next evaluated the neurotrophin response following KA, where SE was relatively mild and cell death was limited, and pilocarpine, where SE was more severe and there was widespread cell death. We used Western blot analysis to characterize the total protein levels of the mature and proforms of BDNF and NGF in mouse hippocampi.

**Figure 2.**
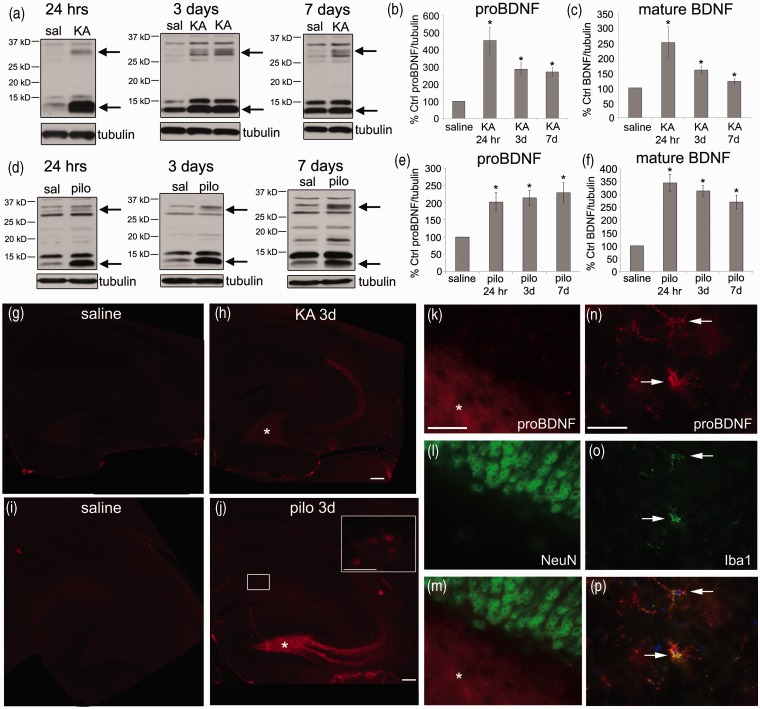
BDNF protein levels are elevated 24 hr following both KA (25 mg/kg) and pilocarpine (320 mg/kg) injection and remain elevated up to 7 days. (a) Representative Western blots of BDNF protein in the hippocampus of saline- and KA-treated animals following 24 hr, 3 days, or 7 days are shown. Anti-tubulin was used to normalize to total protein. Arrows indicate the bands representing proBDNF (upper arrow) and mature BDNF (lower arrow). The graphs represent densitometric analysis of the blots for proBDNF (b) and mature BDNF (c) following KA. Results are shown as % control saline. Data were analyzed by one-way ANOVA followed by Bonferroni. *Significantly different from saline controls (*p* < .05). (KA, *N* = 3 at 24 hr; *N* = 10 at 3 days; *N* = 7 at 7 days). (d) Representative Western blots of BDNF protein in the hippocampus of saline- and pilocarpine-treated animals following 24 hr, 3 days, or 7 days are shown. The graphs represent densitometric analysis of the blots for proBDNF (e) and mature BDNF (f) following pilocarpine. Results are shown as % control saline. Data were analyzed by one-way ANOVA followed by Bonferroni. *Significantly different from saline controls (*p* < .05). (Pilocarpine, *N* = 6 at 24 hr and 3 days; *N* = 3 at 7 days.) (g–j) Horizontal sections from mice 3 days after injection of saline (g,i), KA (h), or pilocarpine (j) stained with an antibody to proBDNF indicating enhanced proBDNF-ir following both KA and pilocarpine. The asterisks in (h and j) indicate proBDNF-ir in the hilus where mossy fibers originate. The inset in (j) shows the boxed area at higher power. Scale bars represent 100 µm. (k–m) Sections from mice 3 days after injection with pilocarpine were double labeled with antibodies to proBDNF (k) and NeuN (l) to show proBDNF expression in mossy fibers in the hilus (indicated with an asterisk) and not neuronal cell bodies in the granule cell layer. Merged image is shown in panel M. Scale bar represents 50 µm. (n–p) Sections from mice 3 days after injection with pilocarpine were double labeled with antibodies to proBDNF (n) and Iba1 (o) to show proBDNF expression in microglia in stratum radiatum. Merged image is shown in (p) with DAPI (blue). Arrows indicate cells ir for both proBDNF and Iba1. Scale bar represents 50 µm. Immunofluorescent images are representative from an *N* of 3 mice per condition.

Wild-type 129SvJ mice were injected with saline, KA (25 mg/kg), or pilocarpine (320 mg/kg; these animals also did not have electrodes implanted) to induce SE and sacrificed 24 hr, 3 days, or 7 days later for hippocampal dissection. For both KA- and pilocarpine-induced seizures, levels of mature BDNF (13 kD) protein were significantly elevated as early as 24 hr and remained elevated up to 7 days after treatment (Figure 2(a), (c), (d), (f)) consistent with the well-established induction of BDNF mRNA and protein following SE (McNamara and Scharfman, 2012). In addition, proBDNF (32 kD) protein levels were significantly upregulated following both KA and pilocarpine at all timepoints (Figure 2(a), (b), (d), (e)). To localize proBDNF in the hippocampus following seizures, we performed immunocytochemistry using an antibody specific for the prodomain of BDNF (Genecoepeia). ProBDNF-ir was clearly elevated following both KA and pilocarpine (Figure 2(g) to (j)) and appeared to be mainly localized to the mossy fiber pathway. Co-expression of proBDNF and NeuN showed diffuse proBDNF-ir in mossy fibers and no proBDNF-ir in the granule cell bodies following pilocarpine (Figure 2(k) to (m)). In addition, proBDNF-ir appeared to be expressed in glial cell bodies following pilocarpine (Figure 2(j), boxed area). Co-expression of proBDNF-ir and Iba1-ir confirmed that proBDNF was localized to microglia throughout the hippocampus following pilocarpine (Figure 2(n) to (p)). This localization in microglia was absent in KA-injected animals. ProBDNF-ir was not found in GFAP-ir astrocytes following pilocarpine (Suppl Figure 1).

**Figure 3.**
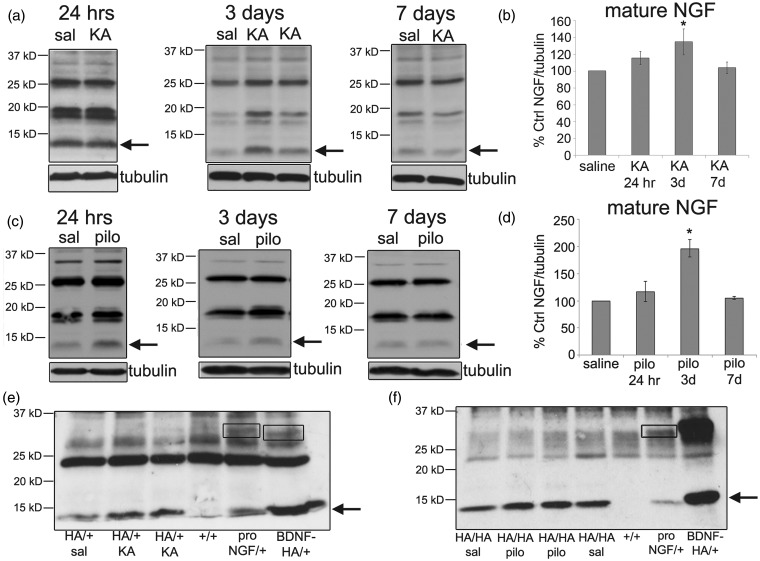
NGF protein levels are significantly elevated following KA (25 mg/kg) or pilocarpine (320 mg/kg) injection. (a) Representative Western blots of NGF protein in the hippocampus of saline- and KA-treated animals following 24 hr, 3 days, or 7 days are shown. Anti-tubulin was used to normalize to total protein. Arrows indicate the bands representing mature NGF. (b) The graph represents densitometric analysis of the blots for mature NGF following KA. Results are shown as % saline control. Data were analyzed by one-way ANOVA followed by Bonferroni. *Significantly different from control (*p* < .05). (KA, *N* = 3 at 24 hr; *N* = 10 at 3 days; *N* = 7 at 7 days.) (c) Representative Western blots of NGF protein in the hippocampus of saline-and pilocarpine-treated animals following 24 hr, 3 days, or 7 days are shown. (d) The graph represents densitometric analysis of the blots for mature NGF following pilocarpine. Results are shown as % saline control. Data were analyzed by one-way ANOVA followed by Bonferroni. *Significantly different from control (*p* < .05). (Pilocarpine, *N* = 6 at 24 hr and 3 days; *N* = 3 at 7 days.) (e, f) Western blot of HA protein in the hippocampus of saline-, KA- (e), and pilocarpine- (f) treated NGF-HA animals following 3 days. Hippocampal lysates were immunoprecipitated with a polyclonal anti-HA antibody and immunoblotted with a monoclonal anti-HA antibody. *N* = 3 at 3 days for KA and pilocarpine. ProNGF-HA/+ and BDNF-HA/+ hippocampal lysates were used as a positive control to document the molecular mass of endogenously expressed proNGF and proBDNF. Non-HA (+/+) lysates were used as a negative control. Boxes surround bands representing proNGF and proBDNF. An arrow indicates the band representing mature NGF and BDNF.

Levels of mature NGF (13 kD) protein were significantly elevated 3 days following both KA or pilocarpine treatment (Figure 3(a) to (d)). To enhance detection of proNGF in hippocampal lysates, we utilized knock-in mice in which the endogenous alleles of the *Ngf* gene were replaced with the *Ngf* sequence containing an HA tag at the C-terminus (NGF-HA mice; (Siao et al., 2012)). In this way, we were able to immunoprecipitate hippocampal lysates from saline-, KA-, or pilocarpine-treated NGF-HA mice with antibodies to HA and immunoblot for HA. As positive controls to document the molecular mass of endogenously expressed proNGF or proBDNF, hippocampal lysates from BDNF-HA and proNGF-HA mice were used (Yang et al., 2009; Siao et al., 2012). BDNF-HA mice were knock-in mice in which the *Bdnf* sequence contains an HA tag at the C-terminus. As previously demonstrated (Yang et al., 2009), immunoprecipitation with an HA antibody followed by immunoblot with an HA antibody revealed bands at 14 and 32 kD that correspond to mature and proBDNF (Figure 3(e)), indicating that the HA immunoprecipitation is effective in identifying both mature and proneurotrophins. ProNGF-HA mice were knock-in mice in which one allele contains a cleavage-resistant *Ngf* sequence with an HA tag at the C-terminus, resulting in overexpression of proNGF (Siao et al., 2012). As shown in Figure 3(e) and (f), HA immunoprecipitation detected a 34-kD proNGF isoform in proNGF-HA/ + lysates, indicating that we can detect proNGF using HA immunoprecipitation. Although mature NGF was present, proNGF was undetectable by HA immunoprecipitation in NGF-HA hippocampal lysates following saline, KA, or pilocarpine injection (Figure 3(e) and (f)), indicating that proNGF was not elevated following SE in mice. These data are different than what has been reported using pilocarpine in rats (Volosin et al., 2008) and indicate a species difference in response to SE. Although proNGF is not elevated, proBDNF is elevated in the mouse hippocampus following seizures and therefore indicate that proBDNF may play a role in seizure pathology in mice.

### p75^NTR^ Protein Levels Are Transiently Elevated Following Both KA and Pilocarpine in the Hippocampus

p75^NTR^ is upregulated following many injury paradigms, including seizures (Friedman, 2010). The best characterized role for p75^NTR^ following injury is to induce apoptosis, particularly in response to proneurotrophins (Beattie et al., 2002; Troy et al., 2002; Harrington et al., 2004). Therefore, we evaluated changes in p75^NTR^ following KA- or pilocarpine-induced SE.

**Figure 4.**
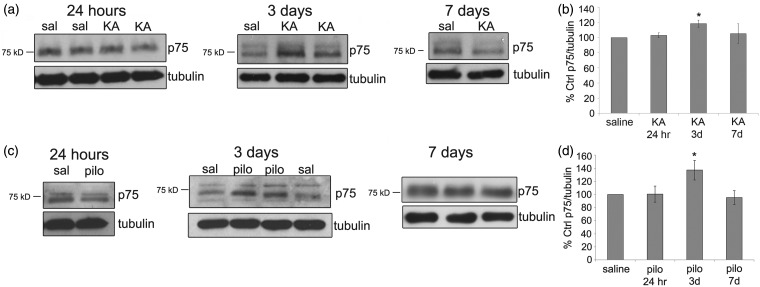
p75^NTR^ protein levels are acutely elevated 3 days following both KA (25 mg/kg) and pilocarpine (320 mg/kg) injection. (a) Representative Western blots of p75^NTR^ protein in the hippocampus of saline- and KA-treated animals following 24 hr, 3 days, and 7 days are shown. Anti-tubulin was used to normalize to total protein levels. (b) The graph represents densitometric analysis of the blots following KA. Results are shown as % saline control. Data were analyzed by one-way ANOVA followed by Bonferroni. *Significantly different from control at *p* < .05. (KA, *N* = 3 at 24 hr; *N* = 9 at 3 days; *N* = 8 at 7 days.) (c) Representative Western blots of p75^NTR^ protein in the hippocampus of saline- and pilocarpine-treated animals following 24 hr, 3 days, and 7 days are shown. (d) The graph represents densitometric analysis of the blots following pilocarpine. Results are shown as % saline control. Data were analyzed by one-way ANOVA followed by Bonferroni. *Significantly different from control at *p* < .05. (Pilocarpine, *N* = 6 at 24 hr and 3 days; *N* = 3 at 7 days.)

**Figure 5.**
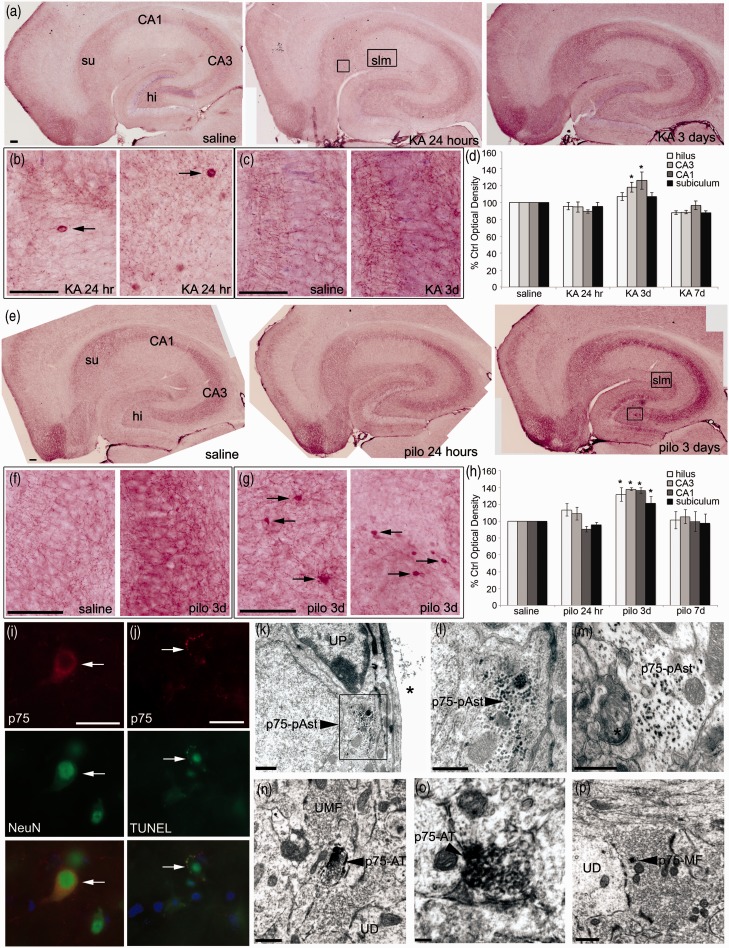
p75^NTR^-ir is upregulated in the hippocampus 3 days following both KA (25 mg/kg) and pilocarpine (320 mg/kg) injection. (a) Representative p75^NTR^-labeled horizontal sections are shown at low power following 24 hr and 3 days KA. Areas of the hippocampus are labeled in the saline panel, hi = hilus and su = subiculum. (b) Higher power images of the boxed areas in (a) 24 hr after KA. Arrows indicate p75^NTR^-ir cell bodies in stratum lacunosum-moleculare (slm). (c) Higher power images of area CA3 3 days following saline (left panel) or KA (right panel) indicating enhanced p75^NTR^-ir in fibers. Scale bars represent 100 µm. (d) Graph represents densitometric analyses of p75^NTR^-ir following KA. Data were analyzed by one-way ANOVA followed by Bonferroni. *Significantly different from control at *p* < .05. *N* = 3 mice at each time point. (e) Representative p75^NTR^-stained horizontal sections are shown at low power following 24 hr and 3 days pilocarpine. (f) Higher power images of area CA3 3 days following saline (left panel) or pilocarpine (right panel) indicating enhanced p75^NTR^-ir in fibers. (g) Higher power images of the boxed area in (e) 3 days after pilocarpine. Arrows indicate p75^NTR^-ir cell bodies in the hilus (left panel) and slm (right panel). Scale bars represent 100 µm. (h) Graph represents densitometric analyses of p75^NTR^-ir following pilocarpine. Data were analyzed by one-way ANOVA followed by Bonferroni. *Significantly different from control at *p* < .05. *N* = 3 mice at each time point. (i) Sections from mice 3 days after pilocarpine were double labeled with antibodies to p75^NTR^ (red) and NeuN (green). An arrow indicates a cell expressing both p75^NTR^ and NeuN. DAPI (blue) is shown in the merged image. Scale bar represents 50 µm. (j) Sections from brains of pilocarpine injected mice were processed for TUNEL labeling (green) and stained with an antibody for p75^NTR^ (red). Double labeling indicates apoptosis in cells stained for p75^NTR^ (shown with an arrow). DAPI (blue) is shown in the merged image. Scale bar represents 50 µm. Immunofluorescent images are representative from an *N* of 3 mice per condition. (k–m) Immuno-EM was used to characterize p75^NTR^-ir cell bodies in slm. p75^NTR^-ir cells were found near blood vessels (k) as well as further away (m). Asterisk in (k) indicates a blood vessel. Asterisk in (m) indicates a synapse. Arrowhead indicates areas of p75^NTR^-ir. The boxed area in (k) is shown at higher magnification in (l). UP = unlabeled pericyte, pAst = protoplasmic astrocyte. Scale bars represent 500 nm. (n–p) Immuno-EM was used to visualize the localization of p75^NTR^ in stratum lucidum of area CA3 in mice (*N* = 3). Arrowheads indicate p75^NTR^-ir in small axon terminals at low (n) and high power (o), confirming that p75^NTR^ is primarily presynaptic. (p) An arrowhead indicates p75^NTR^-ir in a dense core vesicle in a subset of mossy fibers. AT = axon terminal, UD = unlabeled dendrite, UMF = unlabeled mossy fiber. Scale bar in panels (n) and (p) represents 500 nm and scale bar in panel (o) represents 100 nm.

p75^NTR^ protein levels were significantly elevated at 3 days following both KA and pilocarpine and then returned to control levels by 7 days (Figure 4). To confirm the Western blot results, we used immunohistochemistry to localize p75^NTR^ in the hippocampus. P75^NTR^ is highly expressed in the developing brain but is maintained at much lower levels in the adult (Roux and Barker, 2002; Woo et al., 2005; Yang et al., 2009). Horizontal sections from saline-, KA-, or pilocarpine-injected mice following 24 hr, 3 days, or 7 days were stained with an antibody to p75^NTR^, and the intensity of immunostaining was measured using Image J. Consistent with studies in rats (Dougherty and Milner, 1999), p75^NTR^-ir was low in fibers in the hippocampus of saline control mice, particularly in area CA3 and the hilus of the hippocampus (Figure 5(a) and (e)). Twenty-four hours following KA, a small number of p75^NTR^-labeled cell bodies were found in stratum lacunosum-moleculare of the hippocampus (Figure 5(b)). However, the intensity of p75^NTR^-ir in the fibers was similar to saline controls after 24 hr (Figure 5(d)). At 3 days following KA, there was a dramatic increase of the intensity of p75^NTR^-ir in the hippocampus compared with saline controls (Figure 5(d)). The enhanced ir appeared to be localized to fibers in the hippocampus, as can be seen in area CA3 (Figure 5(c)). By 7 days following KA, p75^NTR^-ir returned to control levels (Figure 5(d)). Following pilocarpine, the intensity of p75^NTR^-ir was similar to saline controls after 24 hr and enhanced following 3 days (Figure 5(e) and (h)). The enhanced ir following pilocarpine was again localized to fibers, particularly in area CA3 (Figure 5(f)). In contrast to KA, a small number of p75^NTR^-labeled cell bodies were present in the hilus 3 days after pilocarpine (Figure 5(g), left panel). There were also smaller p75^NTR^-labeled cell bodies present near the fissure in stratum lacunosum-moleculare of CA1 3 days following pilocarpine (Figure 5(g), right panel). As with KA, the intensity of p75^NTR^-ir returned to control levels by 7 days following pilocarpine (Figure 5(h)). These data correlate with the increase in total p75^NTR^ protein levels seen by Western blot and indicate that p75^NTR^ is transiently induced in fibers of the hippocampus at 3 days following KA or pilocarpine and in a subset of cell bodies 3 days following pilocarpine.

To characterize the cells in which p75^NTR^ is induced following pilocarpine, we performed double immunofluorescence with p75^NTR^ and both neuronal and glial markers. In the hilus, we detected p75^NTR^ expression in NeuN + neurons (Figure 5(i)). The neuronal p75^NTR^-labeled cell body staining in the pilocarpine animals is similar to previous reports (Roux et al., 1999; Volosin et al., 2008; Bischoff et al., 2012). In addition, TUNEL labeling indicated that there were p75^NTR^-labeled cell bodies in the hilus undergoing apoptosis (Figure 5(j)), which has also been reported in rats (Roux and Barker, 2002; Volosin et al., 2008). The cell bodies present near the fissure in stratum lacunosum-moleculare did not co-label with NeuN (Suppl Figure 2(A)), suggesting they were not neurons. They also did not co-label with GFAP or Iba1 (Suppl Figure 2(B) and (C)), suggesting they were not GFAP + astrocytes or microglia. To more closely examine the identity of the p75^NTR^-ir cell bodies in stratum lacunosum-moleculare following pilocarpine, we utilized immuno-EM. Many of the p75^NTR^-ir cell bodies were found near blood vessels, extending endfeet toward unlabeled pericytes (Figure 5(k) and (l)). In addition, p75^NTR^-ir cell bodies were found separate from blood vessels and surrounding synapses (Figure 5(m)). These cells had the morphological characteristics of protoplasmic astrocytes (gray matter astrocytes): The nucleus exhibited homogeneous karyoplasm with condensations of chromatin beneath the nuclear envelope, the cytoplasm lacked bundles of glial filaments, and the outline of the cell body was very irregular so that it fit into the contours of the elements of the surrounding neuropil (Peters et al., 1991). As GFAP labels only a subset of astrocytes in the brain (Cahoy et al., 2008), this may account for the lack of p75^NTR^ and GFAP co-labeling. Collectively, these data indicate that p75^NTR^ is present in both neurons and a distinct population of GFAP-negative astrocytes following pilocarpine.

In rats, p75^NTR^-ir in the hippocampus is primarily found in axons and axon terminals but is also present in a small number of dendrites (Dougherty and Milner, 1999). To examine the localization of p75^NTR^ in fibers in the mouse hippocampus, we utilized immuno-EM. We focused on stratum lucidum and stratum radiatum of the CA3 region and categorized p75^NTR^-labeled profiles following 3 days saline or pilocarpine injection. As has been reported in rats, p75^NTR^ was mainly localized to presynaptic axon terminals and axons but was also present in a small proportion of dendrites in both subregions of CA3 (Table 1; Figure 5(n) and (o)). However, contrary to what has been reported in rats, a small proportion of mossy fiber terminals contained p75^NTR^-ir (Table 1; Figure 5(p)). After pilocarpine-induced seizures, neither the number nor percentage of the types of profiles containing p75^NTR^-ir changed (Table 1), indicating that the increase in p75^NTR^-labeled fibers seen by light microscopy is not due to the aberrant expression of the receptor.

**Table 1.** Percent of p75^NTR^-Labeled Profiles.

Region	Axon terminal	Axon	Mossy fiber	Dendrite	Total number of p75NTR + profiles evaluated
CA3—stratum lucidum					
Saline	28.9 ± 4.3	34.6 ± 6.2	9.5 ± 3.8	13.7 ± 3.5	70.3 ± 6.3
Pilocarpine	33.5 ± 7	29.1 ± 3.4	11.8 ± 2.3	17.2 ± 2.9	67.7 ± 2
CA3—stratum radiatum					
Saline	38.2 ± 1.7	33.6 ± 6.6		11.8 ± 6.4	36.7 ± 1.4
Pilocarpine	43.1 ± 1.9	38.2 ± 5.5		10.8 ± 7.6	34 ± 4.7

*Note.* p75^NTR^ localization is unchanged 3 days after pilocarpine injection. p75^NTR^-labeled profiles were counted in random EM images of stratum lucidum (15) or stratum radiatum (8) of CA3 from three saline and three pilocarpine-treated mice. Data are presented as the % of the total number of p75^NTR^-positive profiles for each animal ± SE. The total number of p75^NTR^-positive profiles evaluated are shown.

### Sortilin Protein Levels Do Not Change Following KA or Pilocarpine, Whereas sorCS2 Protein Levels Are Elevated in the Hippocampus, but Only Following Pilocarpine

**Figure 6.**
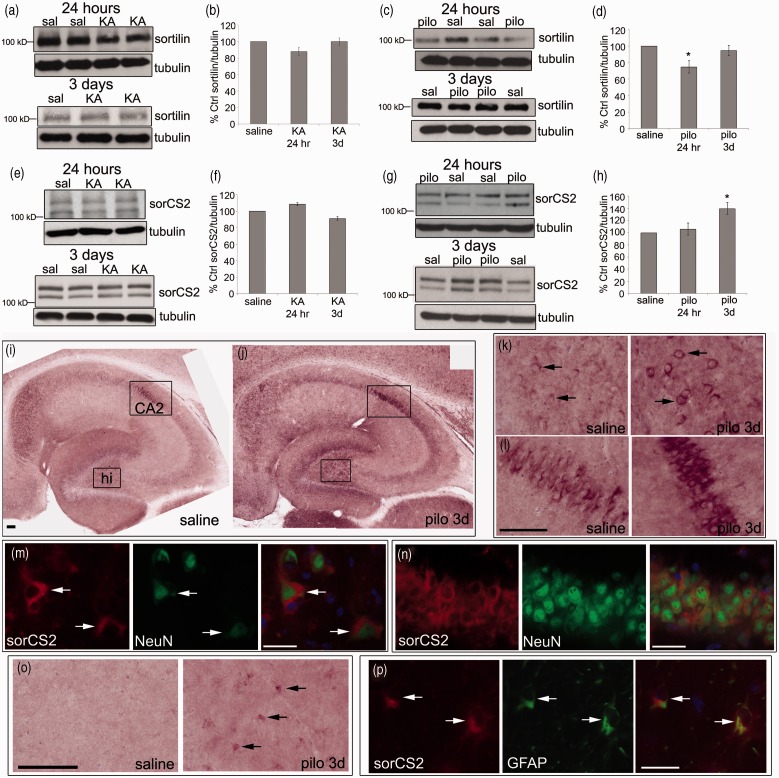
Sortilin and sorCS2 protein levels are unchanged following KA (25 mg/kg), whereas sortilin levels are transiently decreased and sorCS2 levels are elevated 3 days following pilocarpine (320 mg/kg). (a) Representative Western blots of sortilin in the hippocampus of saline- and KA-treated animals following 24 hr and 3 days are shown. Anti-tubulin was used to normalize to total protein. (b) The graph represents densitometric analysis of the sortilin blots following KA. (c) Representative Western blots of sortilin in the hippocampus of saline- and pilocarpine-treated animals following 24 hr and 3 days are shown. (d) The graph represents densitometric analysis of the sortilin blots following pilocarpine. (e) Representative Western blots of sorCS2 in the hippocampus of saline- and KA-treated animals following 24 hr and 3 days are shown. (f) The graph represents densitometric analysis of the sorCS2 blots following KA. (g) Representative Western blots of sorCS2 in the hippocampus of saline- and pilocarpine-treated animals following 24 hr and 3 days are shown. (h) The graph represents densitometric analysis of the sorCS2 blots following pilocarpine. Results are shown as % saline control. Data were analyzed by one-way ANOVA followed by Bonferroni. *Significantly different from control at *p* < .05. (KA, *N* = 3 at 24 hr; *N* = 5 at 3 days. Pilocarpine, *N* = 4 at 24 hr and 3 days.) (i–j) Horizontal sections from brains of saline (i) or pilocarpine (j) injected mice were stained with an antibody for sorCS2. Representative sorCS2-stained sections are shown at low power 3 days after pilocarpine. SorCS2-ir areas are labeled in (I), hi = hilus. Scale bar represents 100 µm. (k) Higher power images of the hilus outlined in (i–j) 3 days after saline (left panel) or pilocarpine (right panel) indicating enhanced sorCS2-ir following pilocarpine. Scale bar represents 50 µm. Arrows indicate cells positive for sorCS2. (l) Higher power images of area CA2 outlined in (i–j) 3 days after saline (left panel) or pilocarpine (right panel) indicating enhanced sorCS-ir following pilocarpine. Scale bar represents 50 µm. (m–n) Sections from mice 3 days after injection with pilocarpine were double labeled with antibodies to sorCS2 (red) and NeuN (green) to show sorCS2 expression in neurons in the hilus (m) and area CA2 (n). Scale bar represents 50 µm. Arrows indicate cells double labeled for sorCS2 and NeuN. DAPI (blue) is shown in the merged images. (o) Higher power images of stratum radiatum after 3 days saline (left panel) or pilocarpine (right panel). Scale bar represents 50 µm. Arrows indicate sorCS2-ir cells with glial morphology. (p) Sections from mice 3 days after injection with pilocarpine were double labeled with antibodies to sorCS2 (red) and GFAP (green) to show sorCS2 expression in astrocytes in stratum radiatum. Scale bar represents 50 µm. Arrows indicate cells double labeled for sorCS2 and GFAP. DAPI (blue) is shown in the merged image. Immunofluorescent images are representative from an N of 3 mice per condition.

Both sortilin and sorCS2 have been reported to act as co-receptors with p75^NTR^ to mediate proneurotrophin actions (Nykjaer et al., 2004; Deinhardt et al., 2011). As we observed elevated levels of proBDNF and p75^NTR^ following SE in mice, we next evaluated changes in sortilin and sorCS2 in the hippocampus. Sortilin protein levels were mostly unchanged following KA and pilocarpine except for a transient decrease 24 hr following pilocarpine (Figure 6(a) to (d)). In contrast, sorCS2 protein levels were significantly elevated 3 days following pilocarpine injection, while no changes were seen following KA injection (Figure 6(e) to (h)). To localize sorCS2 following seizures, we performed immunohistochemistry with a sorCS2 antibody. SorCS2 mRNA is highly expressed in pyramidal cells in area CA2 and the dentate gyrus in the hippocampus (Hermey et al., 2004), and we report the same pattern of sorCS2 immunostaining in saline controls (Figure 6(i)). However, sorCS2-ir was elevated following pilocarpine (Figure 6(j) to (l)). SorCS2 was localized to NeuN-ir cells (i.e., neurons) primarily in the hilus (Figure 6(m)) and area CA2 (Figure 6(n)) of the hippocampus. In addition to neuronal labeling, sorCS2 also appeared in cells that had the morphology of glia, and these were present throughout the hippocampus following pilocarpine (Figure 6(o)). Co-labeling studies with an antibody to GFAP confirmed that sorCS2 was present in astrocytes following pilocarpine (Figure 6(p)). SorCS2 was not expressed in Iba1-ir cells following pilocarpine, suggesting that the expression was weak or absent in microglia (Suppl Figure 3). These data indicate that sorCS2 expression, similar to p75^NTR^, is elevated in both neurons and astrocytes following pilocarpine.

## Discussion

In the current study, we characterized the neurotrophin response in two mouse models of chemically induced SE. KA resulted in limited cell death in the mouse hippocampus while pilocarpine resulted in widespread hippocampal cell death, which correlated with the severity of SE. Following both KA and pilocarpine administration, mature and proBDNF, as well as mature NGF protein levels were significantly elevated, while proNGF protein levels were unaltered, contrary to what has been shown in rat models. ProBDNF was localized to mossy fibers and microglia following pilocarpine. In addition, the intensity of p75^NTR^-ir fibers in the hippocampus was transiently upregulated following both KA and pilocarpine. P75^NTR^ labeling was also upregulated in a population of neurons and astrocytes following pilocarpine. Sortilin protein was unchanged while sorCS2 was upregulated following pilocarpine, but not KA. SorCS2 was localized to neurons as well as astrocytes following pilocarpine. These data indicate that there are significant differences in the proneurotrophin response to seizures between rat and mouse and suggest that there may be different mechanisms by which these ligands impact seizure pathology between the two species. In addition, proBDNF and p75^NTR^ induction are observed independent of cell death, suggesting that these molecules may play other roles following SE.

### Neural Pathology Following Seizures in Mice

Rat models using KA or pilocarpine to induce SE report excitotoxic or apoptotic cell death of pyramidal neurons in areas CA3 and CA1 and dentate hilar neurons in the hippocampus (Turski et al., 1983; Ben-Ari, 1985; Leite et al., 2002; Pitkanen et al., 2006). We report similar pathology following pilocarpine-induced SE in 129SvJ mice, using FJ-B to label degenerating neurons. However, following KA injection, 129SvJ mice had mild SE, and FJ-B labeling showed limited cell loss in the hippocampus following KA, which was probably due to the severity of SE. These data are consistent with the resistance of some mouse strains (e.g., C57Bl/6) to KA-induced cell death in the hippocampus (Schauwecker and Steward, 1997). Because we found that KA injection in mice can elicit only mild SE relative to pilocarpine by EEG, the lack of cell death after KA could be due to the inability to increase the electrical activity sufficiently to initiate excitotoxic mechanisms.

### Differences in the Proneurotrophin Response to Seizures in Mice Versus Rats

In the current work, we report that pro and mature isoforms of BDNF and mature NGF were upregulated following both KA- and pilocarpine-induced seizures in mice, while proNGF was unchanged. In contrast, both proBDNF and proNGF were increased in rats following pilocarpine-induced seizures (Volosin et al., 2008). Moreover, in rats, proNGF was found to play a role in cell death that follows pilocarpine-induced seizures (Volosin et al., 2008). In mice, we found that proNGF was not upregulated following seizures, suggesting that it may not play as important a role. However, proBDNF was robustly elevated and may be an important effector of the seizure pathology. We also found the localization of proBDNF to be different between rats and mice following seizure. In rats, proBDNF was induced in astrocytes in the hippocampus following pilocarpine (Volosin et al., 2008) while we saw proBDNF induction in microglia following pilocarpine in mice.

These differences in the neurotrophin response may reflect species-dependent mechanisms in response to seizure activity. There are multiple mechanisms by which the synthesis and processing of proneurotrophins can be regulated. The availability of extracellular proteases can regulate the cleavage of proneurotrophins into their mature domains. For example, matrix metalloproteinase-7 (MMP-7) can cleave both proBDNF and proNGF (Lee et al., 2001). Following KA-induced seizures in rat, MMP-7 levels are decreased and the inhibitor of MMP-7 activity, tissue inhibitor of metalloproteinase 1 (TIMP-1) levels are increased in the same cells that express proNGF (Le and Friedman, 2012), suggesting that the regulation of these enzymes is responsible for the upregulation of proNGF in rats. As we did not see the upregulation of proNGF, it is possible that this regulation may be different in mice. In addition, neurotrophin synthesis is subject to a high degree of transcriptional and posttranscriptional regulation. Both the BDNF and NGF genes use multiple promoters, which can result in alternative splicing and generation of multiple transcripts (Selby et al., 1987; Aid et al., 2007). Neuronal activity, including seizures, results in increases in the usage of particular BDNF promoters and transcripts (Timmusk et al., 1995) that may regulate the levels and localization of BDNF isoforms. BDNF is also subject to epigenetic regulation particularly following neuronal activity (Tsankova et al., 2004; Cortes-Mendoza et al., 2013). Considering the multiple checkpoints of protein regulation, it is possible that rats and mice may have distinct mechanisms to induce and maintain neurotrophin levels following seizure.

### Upregulation and Localization of p75^NTR^ Following Seizures

Following both KA and pilocarpine, we found acute upregulation of p75^NTR^ in the hippocampus. In rats, p75^NTR^ was upregulated following seizures and a proportion of p75^NTR^-ir cells became apoptotic (Roux et al., 1999; Volosin et al., 2006, 2008). In mice, we and others (Bischoff et al., 2012) have found this to be true following pilocarpine, as p75^NTR^-labeled cell bodies in the hilus were co-labeled with TUNEL, supporting the hypothesis that p75^NTR^ plays a role in cell death. However, we also could find p75^NTR^-labeled cell bodies in stratum laconosum-moleculare of CA1 24 hr following KA and 3 days following pilocarpine. We identify these cells by EM as protoplasmic astrocytes. Astrocytes play an important role in CNS function, both in normal development and following injury (Zhang and Barres, 2010). They support neurons and synapse formation and function as well as regulate the blood–brain barrier. We found p75^NTR^-ir astrocytes extending processes toward both blood vessels and synapses following pilocarpine. P75^NTR^ has also been detected in astrocytes in the hippocampus following seizures in rat (Cragnolini et al., 2009) and plays a role in the proliferation of astrocytes in response to neurotrophins (Cragnolini et al., 2009, 2012). The function of p75^NTR^ in processes distinct from cell death is still unclear and warrants further investigation, particularly following injury.

Interestingly, we found that p75^NTR^ was transiently upregulated in fibers in the hippocampus, particularly in areas CA3 and CA1, following both KA and pilocarpine. By EM, we were able to confirm that these p75^NTR^-labeled fibers were mostly axons and axon terminals. However, we also found that a small number of dendrites and mossy fibers contained p75^NTR^-ir. The number and types of profiles containing p75^NTR^ did not change following seizures, suggesting that the increases we see by light microscopy are not an aberrant expression of the receptor but may reflect an increase in expression already present in the uninjured brain. Our localization of p75^NTR^-ir in the uninjured hippocampus is largely consistent with the work of others evaluating p75^NTR^-ir by light and electron microscopy. Prior reports indicate that p75^NTR^-ir is in fibers throughout the hippocampus (Gomez-Pinilla et al., 1987; Pioro and Cuello, 1990; Dougherty and Milner, 1999). Many of these p75^NTR^-labeled fibers in the hippocampus are cholinergic afferents originating from the basal forebrain, as they exhibit acetylcholinesterase (AChE) and choline acetyltransferase (ChAT) ir (Gomez-Pinilla et al., 1987; Pioro and Cuello, 1990). Following pilocarpine, there is an increased density of AChE stained fibers that correlates with the increase of p75^NTR^-labeled fibers in the inner molecular layer of the dentate gyrus (Holtzman and Lowenstein, 1995), suggesting that the majority of the increased p75^NTR^ expression we observed in fibers following seizures may represent an increase in cholinergic input or expression. However, EM analysis of p75^NTR^-ir in this study and others has localized the receptor to a small number of dendritic shafts and spines within the hippocampus (Dougherty and Milner, 1999; Woo et al., 2005) as well as mossy fibers, suggesting that p75^NTR^ signaling can occur on noncholinergic neurons in the hippocampus as well.

### Expression of Sortilin and sorCS2 Following Seizures

Sortilin can act as a co-receptor with p75^NTR^ to induce neuronal cell death (Nykjaer et al., 2004; Teng et al., 2005) and may play a facilitatory role following injury (Teng et al., 2010; Nykjaer and Willnow, 2012) because levels of sortilin and p75^NTR^ are both upregulated in some injury paradigms (Jansen et al., 2007; Wei et al., 2007; Provenzano et al., 2008; Nykjaer and Willnow, 2012). However, we did not see an elevation of sortilin following KA or pilocarpine in mice. In the rat, sortilin levels were not elevated following pilocarpine-induced seizures, despite elevations in p75^NTR^ (Unsain et al., 2008). However, we did see a significant decrease of sortilin protein levels 24 hr after pilocarpine. This decrease may reflect a change in localization of the sorting receptor. Normally, 90% of sortilin resides in intracellular compartments (Sarret et al., 2003; Chen et al., 2005; Willnow et al., 2008) and has a well-described role in protein trafficking (Lane et al., 2012), including trafficking of BDNF to lysosomes (Chen et al., 2005; Evans et al., 2011). Following injury, it is possible that sortilin is internalized and that the pool of sortilin is reduced due to increased lysosomal trafficking, although formal studies of sortilin trafficking would be needed in the future to address this hypothesis.

SorCS2, a sortilin family member, can interact with proneurotrophins and act in a complex with p75^NTR^ to induce neuronal process retraction (Deinhardt et al., 2011; Anastasia et al., 2013). The mRNA of SorCS1 and sorCS3, related sortilin family members, has been found to increase in mice following KA-induced seizures, while sorCS2 mRNA did not change (Hermey et al., 2004). In this study, we found that sorCS2 protein is upregulated following pilocarpine. In addition, we found sorCS2 was upregulated in both neuronal and glial cells. This upregulation did not occur following KA, the less severe of the two seizure models, indicating that severe damage may be required for sorCS2 upregulation. Formal studies of the role of sorCS2 in neurons and glial cells following injury would aid in understanding the differences between the two seizure models.

### Glial Expression of proBDNF and sorCS2

The roles of proneurotrophins in the CNS have been studied mainly in neurons (Teng et al., 2010). In the uninjured hippocampus, we found proBDNF in mossy fibers, p75^NTR^ in axons and axon terminals, and sorCS2 in neurons in the hilus and area CA2. After SE, this expression pattern in neurons was enhanced. Furthermore, we found that proBDNF was specifically upregulated in microglia, and p75^NTR^ and sorCS2 were upregulated in astrocytes in the hippocampus following pilocarpine. This expression was absent in the uninjured hippocampus. These findings are consistent with the growing appreciation of the role of glia in epilepsy (reviewed in (Binder and Carson, 2013; Devinsky et al., 2013)). We found that microglia may be a source of proBDNF following SE in mice, making these cells potential targets in therapies aimed at ameliorating damage. We also found p75^NTR^ and sorCS2 expressed in astrocytes following SE, indicating that proneurotrophin signaling may occur in these cells as well as neurons following SE. p75^NTR^ does not influence cell death in astrocytes but may regulate their proliferation in response to neurotrophins (Cragnolini et al., 2009). Astrocytes play a variety of roles in maintaining the function of the CNS (Barres, 2008), and so the function of p75^NTR^ and sorCS2 in astrocytes following SE warrants further investigation.

## Conclusion

There are several mechanisms that can contribute to the development and pathology of human epilepsy and neurotrophins are one family of factors that may contribute to epileptogenesis (Binder et al., 2001; Friedman, 2010; McNamara and Scharfman, 2012), although their contributions to pathology are still unclear. What makes their role more complex is the finding that pro as well as mature isoforms of the neurotrophins have distinct functions in CNS development and following injury. ProNGF is an important mediator of cell death following seizures in rat (Volosin et al., 2008). We find proNGF is not induced in the hippocampus following SE in mice, indicating distinct mechanisms for neurotrophin induction between the species. However, proBDNF and p75^NTR^ are elevated in our mouse models, whether or not cell death is present. Another potential mediator of proneurotrophin action, sorCS2, is also elevated following SE in mice. It is important to understand the differences between rat and mouse in the neurotrophin response so that future work can use the genetic models necessary to further dissect the actions of mature/pro-neurotrophins following seizures.
